# Treatment of heart failure in adults with thalassemia major: response in patients randomised to deferoxamine with or without deferiprone

**DOI:** 10.1186/1532-429X-15-38

**Published:** 2013-05-20

**Authors:** John B Porter, John Wood, Nancy Olivieri, Elliott P Vichinsky, Ali Taher, Ellis Neufeld, Patricia Giardina, Alexis Thompson, Blaine Moore, Patricia Evans, Hae-Young Kim, Eric A Macklin, Felicia Trachtenberg

**Affiliations:** 1 University College London, London, UK; 2 Children’s Hospital of Los Angeles, Los Angeles, USA; 3 Toronto General Hospital, University Health Network, Toronto, ON, Canada; 4 Children’s Hospital & Research Center Oakland, Oakland, CA, USA; 5 American University of Beirut, Beirut, Lebanon; 6 Children’s Hospital, Boston, MA, USA; 7 Weill Medical College of Cornell University, New York, NY, USA; 8 Children’s Memorial Hospital, Chicago, IL, USA; 9 National Heart, Lung and Blood Institute (NHLBI), Bethesda, MD, USA; 10 New England Research Institutes, Watertown, MA, USA; 11 Massachusetts General Hospital, Boston, MA, USA; 12 Department of Haematology, University College London, UCL Cancer Institute, Paul O’Gorman Building, 72 Huntley Street, London WC1E 6BT, UK

**Keywords:** Thalassemia, Heart failure, Deferoxamine, Deferiprone, Combination

## Abstract

**Background:**

Established heart failure in thalassaemia major has a poor prognosis and optimal management remains unclear.

**Methods:**

A 1 year prospective study comparing deferoxamine (DFO) monotherapy or when combined with deferiprone (DFP) for patients with left ventricular ejection fraction (LVEF) <56% was conducted by the Thalassemia Clinical Research Network (TCRN). All patients received DFO at 50–60 mg/kg 12–24 hr/day sc or iv 7 times weekly, combined with either DFP 75 at mg/kg/day (combination arm) or placebo (DFO monotherapy arm). The primary endpoint was the change in LVEF by CMR.

**Results:**

Improvement in LVEF was significant in both study arms at 6 and 12 months (p = 0.04), normalizing ventricular function in 9/16 evaluable patients. With combination therapy, the LVEF increased from 49.9% to 55.2% (+5.3% p = 0.04; n = 10) at 6 months and to 58.3% at 12 months (+8.4% p = 0.04; n = 7). With DFO monotherapy, the LVEF increased from 52.8% to 55.7% (+2.9% p = 0.04; n = 6) at 6 months and to 56.9% at 12 months (+4.1% p = 0.04; n = 4). The LVEF trend did not reach statistical difference between study arms (p = 0.89). In 2 patients on DFO monotherapy during the study and in 1 patient on combined therapy during follow up, heart failure deteriorated fatally. The study was originally powered for 86 participants to determine a 5% difference in LVEF improvement between treatments. The study was prematurely terminated due to slow recruitment and with the achieved sample size of 20 patients there was 80% power to detect an 8.6% difference in EF, which was not demonstrated. Myocardial T2* improved in both arms (combination +1.9 ± 1.6 ms p = 0.04; and DFO monotherapy +1.9 ± 1.4 ms p = 0.04), but with no significant difference between treatments (p = 0.65). Liver iron (p = 0.03) and ferritin (p < 0.001) both decreased significantly in only the combination group.

**Conclusions:**

Both treatments significantly improved LVEF and myocardial T2*. Although this is the largest and only randomized study in patients with LV decompensation, further prospective evaluation is needed to identify optimal chelation management in these high-risk patients.

## Background

Although cardiomyopathy from iron overload is becoming less common in the early lives of Thalassemia Major (TM) patients, particularly in cohorts born after sc DFO became available from an early age [1], the terminal event for older patients is often iron mediated cardiac disease. Recent reviews highlight the paucity or lack of controlled data comparing different chelation regimens in the management of heart failure and other complications of iron overload [2,3].

Heart failure can sometimes be reversed with continuous DFO monotherapy [4-6] but does not always respond even to intensive 24 h IV DFO monotherapy, particularly in patients with severe cardiac iron loading and T2* values <6 ms by cardiovascular magnetic resonance (CMR) [6]. Several lines of evidence suggest that patients with mild to moderate heart iron and without heart failure respond more effectively to regimes containing DFP compared to DFO monotherapy [7-10]. In a randomised study, mild to moderate myocardial iron (T2* of 8–20 ms) improved more over 1 year with combined DFO and DFP than with standard dose DFO 5 day/week [8]; LVEF, while normal in all cases, also increased more in the patients treated with combined therapy. An observational study also suggests that, when combined with DFP, ‘standard’ discontinuous DFO can improve impaired LV function in patients with T2* values <8 ms [9]. Studies with deferasirox monotherapy, whilst showing clear improvements in myocardial T2*, have focused only on patients with LVEF ≥ 56% [11].

At the time that our study was designed (commencing 2005) and undertaken, DFP was not licensed in North America and the investigators judged that a study comparing established first line monotherapy with DFO with or without the addition of DFP (combination) would be helpful in defining whether addition of DFP conferred further benefit in this setting. In this study, physicians entering patients sought to administer DFO at the maximum safe doses commensurate with body iron loading for as many hours daily as were achievable with addition of DFP (combination arm) or placebo (DFO monotherapy arm).

## Methods

### Study design and aims

This was a 12 month Phase II, multi-site, group-sequential, randomized, double-blind, placebo controlled comparison of combination deferoxamine (DFO) and deferiprone (DFP) vs DFO monotherapy (with placebo) in transfusion-dependent adult TM patients with decreased left ventricular ejection fraction (LVEF). The primary aim was to evaluate whether DFP and DFO combination therapy produced greater improvement in LVEF than with DFO monotherapy. The secondary aims were: (1) To evaluate whether myocardial iron burden, as estimated by myocardial T2*, differed between these treatments. (2) To compare changes in other measures of iron overload such as ferritin and liver iron concentrations (LIC) between the two treatments. (3) To compare safety markers of combined therapy with DFO monotherapy.

Subcutaneous or intravenous DFO was administered daily at 50–60 mg/kg for 12–24 hr/day 7 days/week. When necessary, the DFO dose was adjusted downwards to take account of falling (or low baseline) ferritin so as to minimise the risk of audiometric and retinal toxicity or upwards at the clinician’s discretion for inadequate response. In addition to this DFO regimen, DFP (75 mg/kg/day) or placebo was administered orally in 3 divided doses and timed so that 2 of 3 doses were contemporaneous with the DFO infusion.

The inclusion criteria were: (1) ≥8 transfusions a year, age ≥18 years and LVEF ≤56% by balanced steady state free precession (SSFP) CMR, (2) baseline serum ferritin >1000 μg/L or <1000 μg/L, confirmation of myocardial iron loading by cardiac T2* <20 ms. Exclusion criteria included: contraindication to CMR: severe congestive heart failure (HF) (NYHA Classification IV): current treatment for hepatitis: creatinine clearance <50 ml/min: neutrophils <1.5 × 10^9^/L or platelet count <80 × 10^9^/L: treatment with DFP or deferasirox during the previous 2 weeks or previous adverse experience to DFP: patients unwilling to consider DFO at 50–60 mg/kg 12–24 hours per day 7 days per week.

Participants were from the Thalassemia Clinical Research Network (TCRN). Out of 190 initially contacted, 104 were screened, 30 were eligible, 22 agreed to randomisation, and 20 were randomized and treatment initiated. Patients receiving treatment were from Toronto (6), Boston (4), Oakland (4), Beirut (4), Chicago (1) and New York (1). The study protocol was approved by the TCRN Data and Safety Monitoring Board and by the ethical review boards of all TCRN institutions. Written informed consent was obtained from all participants. An independent safety monitoring committee monitored safety and progress of the trial. The trial ran between 2005 and 2008 when the study was terminated due to slow patient accrual.

### Baseline evaluations

Blood for baseline central analysis of iron assays was drawn before treatment initiation. Cardiac baseline measurements were: Holter monitor, electrocardiogram (EKG), echocardiogram, and 6-minute walk. Results from the screening CMR were considered as baseline measurements. Liver Iron Concentration (LIC) was performed by liver R2 MRI [12], biopsy was performed in 1 patient and SQUID biosusceptometry in 3 patients. Audiometry and ophthalmology evaluations were performed within 90 days before or within 14 days after study treatment initiation.

### Randomization

Participants were randomized within 60 days of CMR screening and assigned in a 1:1 ratio stratified by New York Heart Association Classification. Randomization and stratification was performed by ADEPT (the same system we used for data entry) with a sequence using permuted random blocks. This uses an algorithm for randomization that allows specification of chosen parameters, such as stratification and imbalance tolerance. The parameters for randomization were specified in the protocol. The ADEPT algorithm maintains the imbalance at all times, decreasing statistical problems resulting from stopping the trial before completion. To maintain balance at all sites, a maximum imbalance in allocation of 2 participants was permitted. Treatment was to be initiated within 72 hours of randomization and after completion of all baseline data collection. Treating clinicians were blinded to treatment allocations.

### Follow-up schedule

On-treatment blood counts were performed every 7 days and at each monthly study visit, which included an interview for review of symptoms, compliance, concomitant medications and transfusion data. Every 12 weeks, physical examinations, weight, and fasting chemistries were performed. At 24 and 52 weeks, cardiac function by CMR, cardiac T2* and liver R2 (= 1/T2) by MRI, 24-hr Holter monitor, 6-minute walk, audiometry, and ophthalmology were performed. For participants with serum ferritin <1000 μg/L, audiometry and ophthalmology were performed at weeks 12 and 36 in addition to weeks 24 and 52. Participants for whom study medication was suspended or permanently discontinued (due to serious adverse events) continued to be seen every four weeks following the normal schedule.

### Methodology for LVEF and T2*

All CMR scans were performed at 1.5 T including General Electric (Cornell, Children’s Hospital Boston, Toronto General Hospital, Children’s Hospital Los Angeles), Siemens (Children’s Hospital Philadelphia), and Philips (Children’s Hospital Oakland, American University of Beirut) platforms. Cardiac T2* measurements were collected using either a multiple-echo gradient echo (General Electric and Siemens) or single-echo gradient echo pulse sequence with 8 echoes collected between 2.2 and 18 ms. All sites were required to scan manganese chloride phantom having R2* spanning 30-500 Hz (where R2* = 1/T2*), with good linearity with respect to manganese concentration and good overall R2* agreement (COV <10%). Cardiac volumes and ejection fraction were derived from fifteen short axis steady-state free precession images using the MASS software package (Medis, The Netherlands). Cardiac T2* was derived using validated techniques [13]. Some authors have reported lower T2* values using this fitting algorithm compared with a truncated exponential fit for T2* <5 ms [14] but such differences observed in the core laboratory appear to be no greater than 15% even at T2* below 1 ms [15]. CMR processing was performed at a central core laboratory located at Children’s Hospital Los Angeles.

### Statistical analysis

The intended sample size of 86 patients (N = 43 per arm) had 80% power to detect a 5% difference in LVEF between the two arms after 1 year, assuming a standard deviation of change in LVEF of 7.46%, and 20% loss to follow-up. Variance estimates for the power analysis were based on prior longitudinal studies of TM patients with cardiac dysfunction at University College London Hospitals (UCLH) with planned interim updates to the required sample size based on actual observed variance, using an information-based group sequential trial design. For baseline differences, the *t*-test was used for continuous variables and the fisher’s exact test was used for categorical variables to compare two treatment groups at baseline. This paper considers observed trends in the key data (LVEF, T2*, ferritin, LIC) using the per protocol analysis. Linear mixed models with fixed terms for treatment arm, time, and the treatment x time interaction were used, allowing for random participant-specific intercepts and slopes. Compound symmetric covariance structure was assumed. These mixed models provide unbiased estimates when data are missing at random, conditional on the observed data and model assumptions.

The study was stopped early by NHLBI when analysis of the interim data confirmed a required sample size of close to the original 86 that was not achievable within the required time frame. The achieved sample size had 80% power to detect an 8.6% difference in LVEF. Although the final study sample provides inadequate power for the primary aim, it was considered valuable to compare paired means of the primary and secondary endpoints from the subset of subjects who completed follow-up, as well as the safety measures at available time points.

## Results

### Patient baseline data

Baseline patient characteristics including age, LVEF, cardiac T2*, LIC, serum ferritin and 6 minute walk are shown in Table 1. Baseline echo was also obtained in DFO monotherapy (LVEF = 56.8 ±7.3%) and combination arms (LVEF = 54.7 ±5%) (p = 0.46).

**Table 1:** Baseline values for LVEF, myocardial T2*, LIC, ferritin and 6 minute walk

**Patient**	**Arm**	**Age**	**LVEF**	T2*	**LIC^#^**	**Ferritin**	**6 minute**
		**Sex**	**%**	**ms**	**mg/g/dw**	**μg/L**	**Walk**
			**Base**	**Base**	**Base**	**Base**	**Distance m**
1a	Comb	23, M	51.9	5.1	34.8	5893	526
2a	Comb	30, F	49.9	5.9	4.7	2817	425
3a	Comb	22, M	35.5	2.7	6.7	2961	nd
4a	Comb	30, F	48.9	6.3	12.1	3637	nd
5a	Comb	19, M	38.8	7.1	14.2	3054	nd
6a	Comb	21, M	56.0	21.1	32.1	7125	445
7a	Comb	28, M	55.3	12.3	5.3	1608	550
8a	Comb	33, M	55.2	14.8	9.4	nd	567
9a	Comb	34, M	53.0	10.0	2.9	1962	492
10a	Comb	23, M	53.8	3.3	9.7	2800	389
11a	Comb	28, F	55.4	3.9	7.4	735	359
**Mean**		**26.40**	**51.8**	**8.4**	**12.7**	**3259.2**	**469.1**
SD			5.2	5.7	10.8	1924.8	76.5
1b	Mono	19, F	53.9	12.0	11.7	2623.0	nd
2b	Mono	32, F	42.2	10.7	6.2	864.0	689
3b	Mono	32, M	44.8	2.4	32.1	7394.0	nd
4b	Mono	23, M	51.4	9.0	12.4	4649.0	600
5b	Mono	37, M	46.7	8.3	2.7	2329.0	540
6b	Mono	33, M	54.0	5.9	1.5	280.0	644
7b	Mono	19,F	53.1	3.1	2.7	176.0	390
8b	Mono	17, F	47.7	4.1	6.6	1220.0	287
9b	Mono	20.F	54.9	5.7	12.4	nd	nd
**mean**		**25.80**	**49.9**	**6.8**	**9.8**	**2441.9**	**525.0**
SD			4.6	**3.4**	**9.4**	**2485.1**	**156.1**
p comb		0.52	0.86	0.49	0.54	0.22	0.39
vs mono							

Abbreviations: Comb (combination) = DFP + DFO. Mono (monotherapy) = DFO alone. *SD* = standard deviation of the mean.

# All liver iron measurements were by ferriscan except: arm A patient 5 and 6 arm B patient 4 where SQUID and ARM B patient 3 where liver biopsy was performed.

### Treatments received

The starting dose of DFO and the route given are shown in Table 2. Although the study protocol encouraged clinicians to use 24 h IV DFO where practical, only 1 patient in the combination arm and 2 in the DFO monotherapy arm received close to 24 h iv DFO throughout the study. Two patients in the combination arm received 8–12 h IV DFO. The remainder received sc DFO 7 nights a week at a mean of 12.1 and 12.3 hours/night for combination and DFO monotherapy arms respectively. This represents an increase in duration compared with standard DFO therapy of 8 h. The average DFO starting dose was similar with combination (50 mg/kg) or DFO monotherapy (40 mg/kg). Two patients received only low starting doses in the DFO monotherapy arm <30 mg/kg because of low baseline ferritin (280 and 176 μg/L) but these patients had T2 < 20* confirmed before entering the study.

**Table 2:** Last visit^#^ values for LVEF, myocardial T2*, LIC ferritin and 6 minute walk

**Patient**	**Arm**	**DFO**	**DFO**	**LVEF**	T2*	**LIC**	**Ferritin**	**6 minute**
		**Route**	**Dose**	**%**	**ms**	**mg/g dw**	**μg/L**	**Walk**
		**hr/d**	**mg/kg/d**	**Final**	**Final**	**Final**	**Final**	**Distance m**
1a	Comb	SC,12	52	56.0	5.7	27.7	3628	492
2a	Comb	SC,10	*51*	*56.6*	*6.3*	*nd*	*2747*	554
3a	Comb	IV, 24	*59*	*39.2*	*3.1*	*4.0*	*1154*	378
4a	Comb	SC,12	53	51.3	9.2	2.8	1811	516
5a	Comb	IV, 8	51	58.0	6.0	6.8	3008	352
6a	Comb	SC,12	50	61.1	20.8	16.1	4727	352
7a	Comb	IV, 12	*46*	*59.6*	*19.2*	*2.9*	*343*	550
8a	Comb	SC,13	40	nd	nd	nd	nd	na
9a	Comb	SC,14	44	61.6	20.8	2.1	622	475
10a	Comb	SC,12	50	54.6	3.9	7.7	1130	397
11a	Comb	SC,12	50	65.4	3.5	1.7	200	364
**mean**		**12.9**	**49.6**	**56.3**	**9.8**	**8.0**	**1937.0**	**443**
SD			5.0	7.2	7.4	8.7	1528.0	82.8
1b	Mono	SC, 10	*49*	*50.9*	*11.3*	*14.6*	*4409*	689
2b	Mono	SC,12	60	nd	nd	nd	439	na
3b	Mono	IV, 24	50	nd	nd	nd	v high	na
4b	Mono	IV, 24	*41*	***53.3***	***9.4***	***13.8***	*2540*	590
5b	Mono	SC,14	30	51.6	12.8	1.3	890	563
6b	Mono	SC,14	*23*	66.9	9.9	2.1	413	578
7b	Mono	SC,12	*8*	*49.6*	*3.3*	*3.3*	*152*	410
8b	Mono	SC,12	50	58.2	4.0	2.3	2360	398
9b	Mono	SC,12	50	nd	nd	ns	ns	ns
**mean**		**14.9**	**40.1**	**55.1**	**8.4**	**6.2**	**1600.4**	**538**
SD			16.6	6.5	3.9	6.2	1565.7	112.9

# Last visit uses data obtained at the last visit for each patient : this may be at 12 months, 6 months or before this time. Mean values include patients who completed and who did not complete 12 months of treatment; the latter patients are shown in italics. Statistics are not used from this Table as the pre-planned analysis was on longitudinal data shown in the figures.

Comb (Combination) = DFP + DFO. Mono (monotherapy) = DFO alone. SD = standard deviation of the mean. nd = not done. SC = subcutaneous, IV = intravenous.

### Study completion, dose modifications and AEs

Study accrual began in 2005. A total of 20 subjects had been randomized at the time that the study was stopped in 2008, 11 to the combination arm and 9 to the DFO monotherapy arm. One in the DFO monotherapy arm withdrew before initiating treatment. After early termination, each patient still on study was seen for one last close-out visit, with collection of all 52 week measures, including the primary outcome of CMR. If the subject was within 12 weeks, then the final close-out visit was treated as a 24 or 52 week visit for data analysis. Ten patients completed at least close to 6 months with combination and 6 patients with DFO monotherapy. Seven patients completed at least close to 12 months with combination and 4 patients with DFO monotherapy.

Some patients had both increases and decreases in DFO dosing, depending on ferritin trends and AEs. In the combination arm; DFP was halved and subsequently interrupted in patient 2a due to nausea and vomiting; in patient 3a, DFP was interrupted due to cardiovascular instability (hypotension) and HF at week 4–6; in patient 7, DFP was interrupted due to leucopenia and subsequently stopped at week 19 due to abdominal pain; patient 9a, DFP and DFO was interrupted (week 9–10) due to atrial fibrillation. DFO dosing was decreased in patients 3a, 7a & 11a due to falling ferritin trends with additional mild retinal toxicity in patient 3a. In the DFO monotherapy arm, DFO was dose reduced due to falling serum ferritins in patients 5b, 6b and 8b and the dose later increased in patient 8 as serum ferritin increased. In the DFO monotherapy arm, placebo was interrupted in patient 3b at week 7 due to heart failure.

In total, 17 SAEs were reported with combination (7 ‘at least remotely related’ to treatment) and 6 with DFO monotherapy (2 ‘at least remotely related’ to treatment). The use of the term ‘at least remotely related to’ is a subjective assessment at the choice of the investigator if he/she believes there may be a remote link between treatment and the observed effect. Heart failure developed in 3 patients, one in the combination arm and 2 in the monotherapy arm. Heart failure (HF), in patient 3a receiving combination improved with inotropic support and after removing beta-blockers. HF deteriorated in patients 2b and 3b who both received monotherapy, with death resulting from HF. In patient 2b receiving monotherapy, HF was associated with high fever, nausea and vomiting. In patient 3b receiving monotherapy, was associated with atrial tachycardia and did not improve despite increasing the DFO to 75 mg/kg. Other SAEs with combination arm included high ALT (1), septic shock (1), retinal toxicity (1), line infection (1), meningitis (1), hyperkalemia with hyperglycemia (1), leucopenia (ANC =1.4) (1), atrial fibrillation (1), and urinary infection (1). Other SAEs in the DFO monotherapy arm included pneumonia (1), HF (2), atrial tachycardia (1), high ALT (1) and splenectomy (1).

### Compliance and incomplete dosing

The mean missed days of DFO doses per month were 3.02 in the combination arm and 2.82 in the monotherapy arm. The mean missed doses of DFP were 6.02 per month and 4.51 for placebo in the monotherapy arm. The mean returned pills per month with DFP were 36.3 and 30.0 for monotherapy placebo pills. From missed doses, it can be estimated that 79% of the prescribed DFP was taken throughout the study, compared with 85% of placebo pills in the monotherapy arm. Returned tablets were slightly higher in the DFP group. However patients on DFP or placebo remembered to return tablets on only 59% and 66% of occasions respectively, so that the correlation of missed doses by interview with returned tablets was poor (r = 0.13) unless only patients who returned tablets on more than 80% of occasions were included (r = 0.67). According to trial coordinator/nurse assessments, missed dosing attributable to AEs occurred on 5 assessments with DFP (nausea (3), neutrophil count (1), multiple SAEs (1)) and once with DFO monotherapy. Missed DFO doses were similar in the two arms, equivalent to 90% compliance with no clear difference with IV or SC treatment. Partial dosing was rare. The mean prescribed duration of infusion was only 14 h in the DFP arm and 15 h in the monotherapy arm.

### LVEF changes by CMR

**Figure 1:**
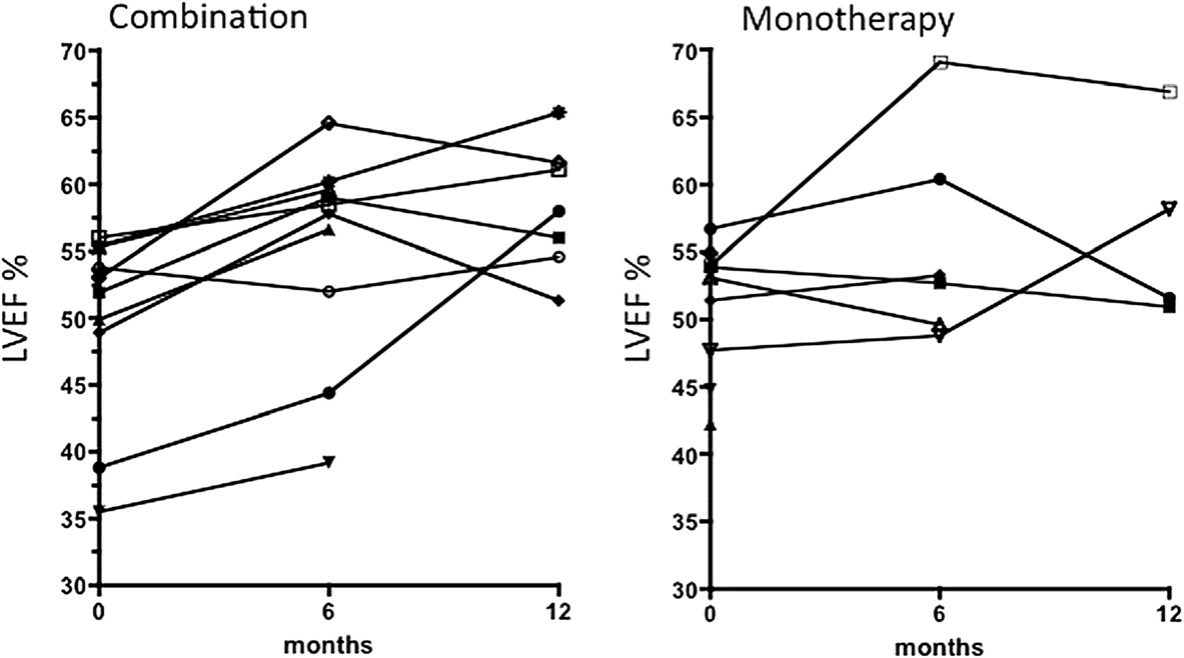
**Baseline, 6 month and 12 month LVEF for each patient in DFO monotherapy and DFP combination arms.** With DFP combination, mean LVEF increased from 49.9% to 55.2% at 6 months (n = 10) and to 58.3% at 12 months (n = 7): with monotherapy LVEF increased from 52.8% to 55.7% at 6 months (n = 6) and to 56.9% at 12 months (n = 4). The improvements are significant over time in both arms (p = 0.04) but without significant difference between arms (p = 0.86 for treatment; p = 0.89 for the interaction).

Figure 1 shows the baseline, 6 month and 12 month LVEF for each patient. Table 1 shows individual baseline and Table 2 final values (clear boxes completed 12 months, shaded completed 6 months). Figure 1 shows that with combination therapy, mean LVEF increased from 49.9% to 55.2% at 6 months (n = 10) and to 58.3% at 12 months (n = 7). With DFO monotherapy, LVEF increased from 52.8% to 55.7% at 6 months (n = 6) and to 56.9% at 12 months (n = 4). Improvement in LVEF was significant in both study arms at 6 and 12 months (p = 0.04), normalizing ventricular function in 9/16 evaluable patients. There was no statistical difference between study arms for treatment (p = 0.86) or for the interaction (p = 0.89). In this context, interaction tests the hypothesis that one group changed more over time than the other, whereas the test for treatment tests for a difference between groups at all times. Neither was significant. The 3 patients who developed heart failure (3a-combination, 2b-monotherapy, 3b-monotherapy) had the lowest baseline LVEF values. Two of these also had very low baseline T2* (2.4 and 2.7 ms) but the third had a moderate T2* of 10.7 ms (Table 1).

### Myocardial iron

**Figure 2:**
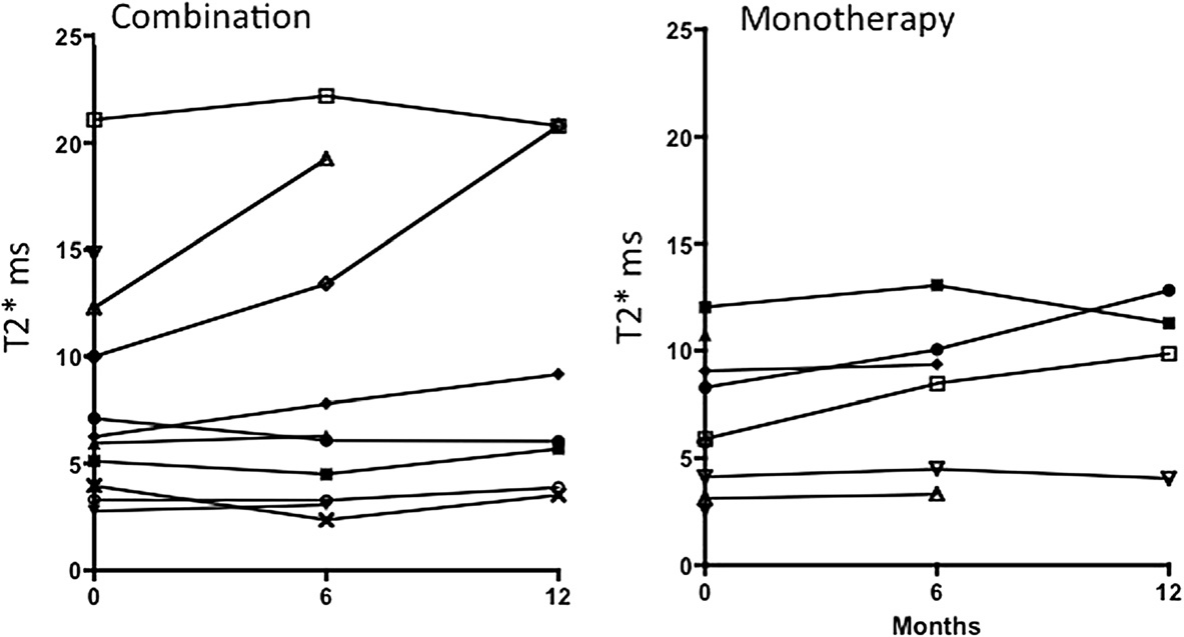
Baseline, 6 and 12 month myocardial T2* values with DFO monotherapy and DFP combination are shown. Myocardial T2* improved in both arms (p = 0.04), with no significant difference between treatments (p = 0.65 for treatment; p = 0.48 for the interaction): improvement with DFP was by 1.5 ms at 6 months and by 1.9 ms at 12 months: with DFP monotherapy this was by 1.1 ms and 1.9 ms respectively.

Figure 2 shows baseline, 6 and 12 month myocardial T2* values. Both treatment arms showed significant improvement over time (p = 0.04). There was no difference between groups (p = 0.65 for treatment; p = 0.48 for the interaction) where tests for treatment, examine for differences between treatment groups at all times whereas tests for interaction, examine whether one group changed more over time than another. At 12 months the mean change in myocardial T2* was nearly identical in the two groups (1.9 ±1.6 ms with combination (n = 7) and 1.9 ±1.4 ms with monotherapy (n = 4).

### Other cardiac assessments

Atrial dysrhythmias and conduction disturbances were common in both treatment arms with no clear progression during the study (other than described as SAEs) and no difference between the two study arms. There were no statistical or clinically significant differences in the 6-minute walk distance (meters, m) in the combination group (baseline 469 m, 6 months 462 m, 12 months 484 m) and the monotherapy group baseline 466 m, 6 months 507 m, 12 months 610 m) and no obvious trends of improvement or deterioration.

### Liver iron (LIC)

**Figure 3:**
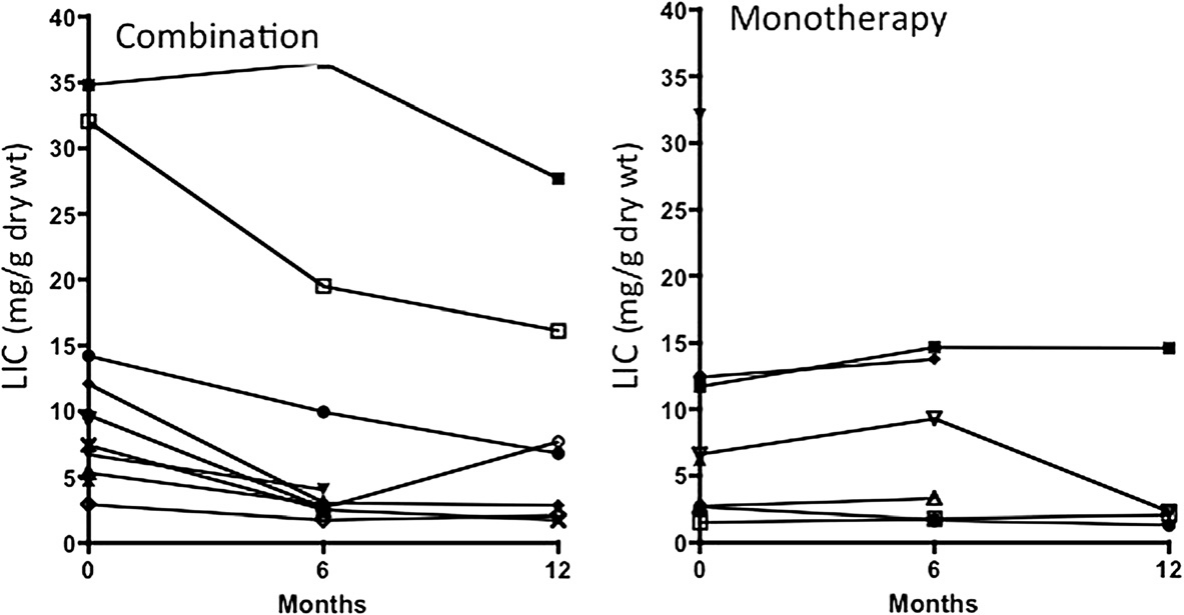
**LIC values at baseline, 6 months and 12 months.** There is a significant decrease in LIC with DFP combination (p = 0.03 for the interaction). At 6 months, there is reduction with DFP combination (from 13.91 ± 3.87 to 9.19 ± 3.91 (n = 9) and a slight increase with DFO monotherapy (from 6.27 ± 1.96 to 7.43 ± 2.44 mg/g dry wt. (n = 6). At 12 months, there is a reduction of 4.29 mg/g dry wt in DFP combination (from 16.1 ± 4.67 to 9.27 ± 3.61 mg/g dry wt, n = 7) and a slight reduction with DFO monotherapy from 5.62 ± 2.2 to 5.08 ± 3.18 mg/g dry wt (n = 4).

Figure 3 shows LIC values at baseline, 6 months and 12 months. LIC time effect p-value was 0.76 and the interaction p-value was 0.03. The interaction p-value of 0.03 in indicates that only DFP combination therapy showed significant decrease over time. The interaction here tests the hypothesis that one group changes more over time than another and shows that LIC decreased more with combination therapy than with monotherapy. LIC declined 4.7 mg/g in patients completing 6 months and 6.8 mg/g in patients completing 12 months of combination therapy. LIC was unchanged in patients treated with DFO monotherapy. The absence of patient follow-up or high baseline LIC values in the DFO monotherapy arm (none >12.4 mg/g dwt) could account for the lack of a significant decrease although baseline LIC did not differ statistically.

### Ferritin

**Figure 4:**
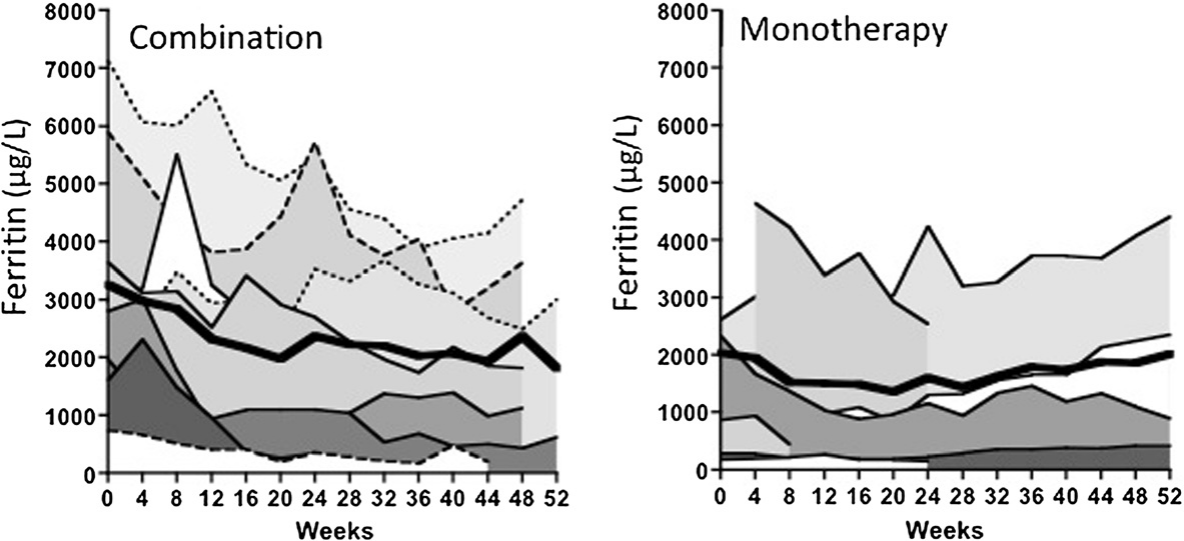
**Baseline and 4 weekly ferritin values in DFP combination and DFO monotherapy arms for individual patients and mean (bold).** With DFP combination, there is a significant downward trend from a baseline of 3308 ± 678 μg/L to 2371 ± 701 μg/L at 6 months (n = 9). Between baseline and 10–12 months, serum ferritin declined from 3601 ± 838 to 2132 ± 646 μg/L (n = 7). In the DFO monotherapy arm, there is a small downward trend from a baseline of 1880 ± 691 μg/L to 1603 ± 636 μg/L at 6 months (n = 6). In 4 samples at baseline and at 12 months, serum ferritin increased from 1613 ± 537 μg/L to 2018 ± 898 μg/L.

In Figure 4, the ferritin trends are shown (with the exception of 1 patient in the DFO monotherapy arm with a baseline ferritin of 7,349 whose ferritin increased to 73,163 μg/L when heart failure developed). At baseline, a higher proportion of patients had ferritin >2500 μg/L in the combination arm (7/11) than monotherapy (3/9). Ferritin trends differed significantly between treatment arms (p < 0.001 for the interaction), with a decrease in ferritin with combination, but an increase with monotherapy, over time.

### Safety markers

Other than transient neutropenia in patient 7a-combination, no agranulocytosis was seen. An early increase in serum creatinine in both study arms of approximately 30% was seen, although values remained within normal limits and trends were not progressive. There was no significant trend in ALT in either arm, although three patients developed sudden increments in ALT (1 DFP, 2 DFO monotherapy). In patient 2a, this rise to 611 iu/L was associated nausea and vomiting and was reported as an AE. In the DFO monotherapy arm, patient 3b developed very high ferritin values (73,163) associated with heart failure as a pre-terminal event. Patient 3b-combination also developed a high ALT also as a pre-terminal event with heart failure. Audiometry, showed worsening change of ≥15 dB on two or more frequencies in only one patient (5a-combination at week 52), which was unilateral and associated with middle ear disease. Eye assessment on patient 3a-combination showed changes consistent with monotherapy induced toxicity requiring DFO dose reduction.

## Discussion

In this study, the intention of the clinicians was to improve treatment either by a combining DFP with DFO or by giving a more intensive DFO regime than prior to the trial, achieved by randomisation to either treatment. DFO was allowed up to 24 h/day and up to 60 mg/kg. The actual dosing allowed clinician discretion, taking into account the risks of DFO overdosing and the practicalities of 24 h DFO IV. Although this study did not recruit the pre-planned patient numbers, it provides valuable information about chelation and response of iron overloaded patients with decreased LV function. This study clearly shows that DFO intensification with or without DFP is effective in improving LVEF and myocardial T2* in this severely affected group of patients. LVEF improvements are less in both study arms than previously reported either with continuous high dose IV DFO monotherapy [4-6] or with combined therapy in TM with established LV dysfunction [8]. This may be because the T2* values in this study at baseline are generally lower than in other reports. For example in a retrospective study of 24 h IV DFO monotherapy for patients with heart failure, the baseline T2* was also low but contained no patients with a T2* <5 ms [6], unlike our study where patients with particularly low T2* values <3 ms were included (Table 1).

Early identification of cardiac risk is key to outcome. Overall, symptomatic heart failure developed in 3 patients, one treated with combination and 2 with DFO monotherapy, one preceded by high fever, the other by nausea, vomiting and arrhythmia. One patient in the combination arm (3a-comb) died within a year of coming off the study, while still on DFP. The numbers of patients entered are too small to conclude whether these differences between treatments are significant however. Recent work has shown that the lower the T2*, the greater the risk of heart failure in the next 12 months which increases as the T2* falls <20 ms, with a relative risk of 159 for T2* <10 ms and of 268 for T2* <6 ms [16]. The T2* technique was introduced to several of the participating centres as part of this study, so that for some patients the T2* was not known prior to the study observations. It is likely that low very T2* values at presentation affect outcome. One death occurred with exceptionally low T2* of 2.4 ms and the patient who died off-study while on combination (3a-Comb) had a baseline T2* of only 2.7 ms. The other death (2b-monotherapy) had a baseline T2* of 10.7 ms and would be predicted as relatively low risk [16] but the deterioration was preceded by severe sepsis, a recognised risk factor in iron overload. At the time of initiation of the study, myocardial T2* was not available as standard of care in all participating centres so that early recognition of high-risk patients on the basis of myocardial T2* was not possible and deterioration in LVEF was therefore the main entry criterion. This selects a very high risk patient group which is still a way in which patients present in centres lacking access to regular myocardial T2* measurement. The introduction of myocardial T2* to some participating centres should help to identify high-risk patients at an earlier point in the future.

With respect to other changes in iron measures, the lack of significant ferritin and liver responses in the DFO monotherapy arm contrasts with previous studies using DFO intensification, possibly reflecting the relatively low baseline LIC and ferritin in this arm. A further important difference between this study and others where LVEF and other improvements were more marked using DFO monotherapy [6,17] is that 24 h treatment was the norm in former studies whereas 24 h treatment was the exception here. Other patient variables such as patient selection, previous chelation history and rapidity of treatment intensification are difficult to control for between studies. The only other report using combined therapy for LV dysfunction, an observational study [9], used similar doses of DFP with discontinuous DFO in the majority of cases. The improvement overall in LVEF at 1 year, from 51% to 63% was greater than in either arm of our study. Changes in ferritin were also greater than in our study, though T2* changes were similar. Poor compliance in our study is unlikely to be a major factor, being close to 80% in the DFP combination arm. The changes in LIC and ferritin in both arms are also less than reported in non-randomized studies with DFO monotherapy [6] or with combined therapy [9].

Tolerability of interventional therapies is better assessed in prospective trials than with retrospective reports. This study did not find significant new issues with tolerability with combined therapy but a larger study would be needed to elucidate whether for example the agranulocytosis increased with intensive combined therapy. ALT changes are previously reported with DFP monotherapy and may also occur with HF. Serum creatinine values showed an early increase of nearly 30% in both study arms and this effect of chelation intensification is of interest because early increments of similar magnitude have been described with deferasirox [18]. Serum creatinine increments have also previously been described with patients commencing DFO at standard doses [19,20] or with intensification of IV DFO [21]. There are no previous reports of serial creatinine measurements with DFP monotherapy or in combination with DFO.

## Conclusions

This study provides randomized prospective information about the efficacy and tolerability of chelation therapies in high-risk patients with reduced LV function, in a field where randomized studies are in short supply [2,3]. Intensification of DFO therapy with or without a DFP combination was effective at improving LV function and myocardial T2*. No difference in response of these measures was demonstrable with the patient numbers studied. While the study is underpowered to show the intended planned difference in LVEF between treatment arms, if a difference of >8.6% had been present there was 80% power to detect this. Thus it is possible that a difference of LVEF improvement between 5–8.6% exists between treatment arms but the study was underpowered to show this. The study does not add any major safety issues in the use of combination under these circumstances so that the decision to add DFP to intensive DFO should be made on a case by case basis, weighing the potential benefits suggested by other studies in less severely affected patients, against the small risk of agranulocytosis with combination therapy. Although this is the largest and only randomized study in patients with LV decompensation, further prospective evaluation with larger patient numbers is needed to identify optimal chelation management in these high-risk patients.

## Competing interests

The lead author has conducted clinical trials on chelation and acted on advisory boards from Novartis and Shire who work in the field of iron chelation.

## Authors’ contributions

JBP co-designed, co-analysed and co-wrote the manuscript. FT co-wrote the manuscript and provided statistical oversight. JW oversaw and set up the network for cardiac data analysis, analysed all cardiac data and contributed to manuscript writing. H-YK contributed to statistical analysis. EM worked on the initial powering of the study. Other authors contributed to manuscript and data review and/or to patient recruitment. All authors read and approved the final manuscript.

## Authors’ information

This is publication number 13 for the Thalassemia Clinical Research Network (TCRN).
